# Surgical rehabilitation of cleft lip and/or palate: evaluation of the Brazilian public health system

**DOI:** 10.1016/j.bjorl.2022.05.008

**Published:** 2022-06-08

**Authors:** Denise Maria M. L. da Silveira, Daniella Reis B. Martelli, Verônica Oliveira Dias, Mário Sérgio Costa da Silveira, Ianná Luana Freitas Almeida, Hercílio Martelli Júnior

**Affiliations:** a Universidade Estadual de Montes Claros (Unimontes), Programa de Pós-Graduação em Cuidado Primário em Saúde, Montes Claros, MG, Brazil; b Secretaria de Saúde do Estado de Minas Gerais, Superintendência Regional de Saúde de Montes Claros, Montes Claros, MG, Brazil; c Universidade Estadual de Montes Claros (Unimontes), Curso de Odontologia, Montes Claros, MG, Brazil; d Instituto Federal do Norte de Minas Gerais, Montes Claros, MG, Brazil; e Universidade Estadual de Montes Claros (Unimontes), Faculdade de Medicina, Montes Claros, MG, Brazil

**Keywords:** Mouth abnormalities, Cleft palate, Cleft lip, Healthcare financing

## Abstract

**Objective:**

To evaluate the surgical rehabilitation of cleft lip and/or palate by the Brazilian public health system.

**Methods:**

Retrospective, analytical and comparative ecological study, with information on hospital procedures performed on individuals with cleft lip and/or palate in Centers authorized by the Brazilian public health system, between the years 2008 and 2020. The information was collected in databases Ministry of Health data.

**Results:**

Between 2008 and 2020, there was an increase of 8 (36.4%) qualified Centers in Brazil, currently having 30 Centers in 100% of the geographic regions. The surgical procedures performed totaled 68,716; with multiple surgeries being the most frequent. Complete cleft lip and palate was the most frequent type in hospital admissions. The public financial resources invested in the surgical rehabilitation of cleft lip and palate in the qualified Lip and Palate Malformation Treatment Centers were US$ 39,693 million, making an average value per procedure of US$ 577.64.

**Conclusions:**

In Brazil, public health system performed and financed, over the years 2008 and 2020, an important volume of surgical procedures for cleft lip and/or palate, which presented a polarization in the Southeast region but with a slight tendency expansion to other regions of the country. The most performed surgical procedures were multiple surgeries and mostly for individuals with cleft lip and palate. The amounts paid showed a heretogeneous distribution in the national territory.

**Level of evidence:**

Level 5: Report containing program evaluation data.

## Introduction

Oral clefts are among the most prevalent congenital abnormalities in humans, comprising an important public health problem.^1^, ^2^ Cleft Lip and/or Palate (CL/P) occur in 70% of cases in non-syndromic form.^3^, ^4^, ^5^ Clinically, CL/P can be classified into Cleft Lip (CL), Cleft Palate (CP), and Cleft Lip and Palate (CLP),^4^, ^6^ and can also be categorized based on the state of the alveolus including the Cleft Lip with Alveolus (CLA); Cleft Lip, Alveolus, and Palate (CLAP); Ceft Lip and Palate with intact alveolus (CLP).^7^

The etiology of Non-Syndromic CL/P (NSCL/P) results from the interaction of genetic, epigenetic, and environmental factors; however, the mechanisms involved are still poorly known.^1^, ^4^, ^8^, ^9^, ^10^ Their prevalence is 1 in 500‒2000 live births, varying according to geographic location, ethnicity, and population socioeconomic level.^8^, ^11^, ^12^ In Brazil, studies on the prevalence of NSCL/P are scarce and vary considerably. Brazilian researches have shown a prevalence ranging from 0.19 and 0.58 per 1000 live births.^13^, ^14^

Rehabilitation of patients with CL/P requires clinical procedures and care, initiated by surgical correction, followed by multidisciplinary procedures in several areas.^8^, ^15^ Aiming at favorable results in phonation and swallowing, it is recommended that surgical correction of the CL be performed in the first 12 months of life and CP up to the age of 18 months.^16^

In Brazil, the surgical treatment of CL/P is provided by a public service, called the Brazilian Unified Health System (SUS) in Lip and Palate Malformation Treatment Centers authorized and organized by the Reference Network for the Treatment of Craniofacial Deformities. These treatment centers must have adequate physical structures and multidisciplinary services, and offer outpatient and in-hospital clinical care, following defined the regulations.^17^ The SUS assistance is universal and free of charge, and its organizational principles include the regionalization and hierarchy of health services. The management and financing of the SUS are the responsibility of all three levels of government: federal, state, and municipal.^18^

Thus, considering the importance of the surgical correction of CL/P in improving the quality of life of individuals,^19^, ^20^ the cost-benefit ratio of surgical treatment^21^, ^22^ and the possible differences present in the provision of surgical treatment in Brazil for having a large territorial extension,^23^ this study aimed to evaluate the surgical rehabilitation of CL/P by the SUS.

## Methods

This was an ecological, retrospective, analytical, and census-based study, with information on hospital procedures performed on individuals with CL/P in centers authorized by the SUS, between 2008 and 2020. Information was collected from the Ministry of Health’s database. It began in 2008 when procedures for assisting CL/P were incorporated into the financial limits of the medium and high complexities of Brazilian municipalities. The units of analyses included all treatment centers authorized by the SUS until 2020, registered in the National Registry of Health Facilities System, by location and date of enablement.

The procedures performed and public financial values invested in the surgical treatment of CL/P were obtained from the records of the authorization for hospitalization forms approved by the SUS and available in the Hospital Information System, where the SUS-authorized treatment centers administer the surgical management of all people with CL/P in a hospital environment. Data collection started from the month and year of the service's implementation and ended in December 2020. The data pertaining to services authorized before 2008 were collected from January 2008. The treatment centers were reimbursed by the SUS based on the information registered in this system. As treatment centers can provide outpatient and hospital care, qualified centers that do not provide surgical procedures may exist, but which provide outpatient procedures registered in the Outpatient Information System, which were not used in this study, as our focus was hospital surgical procedures.

The variables collected from the Hospital Information System were month and year, procedure identification code, total hospitalization value, geographic region, federation unit, treatment centers enabled, and code of the 10th revision of the International Statistical Classification of Diseases and Related Health Problems (ICD-10). Considering that the ICD-10 has only three classifications for CL/P (CL, CP, and CLP) and that the Brazilian public hospital registry system uses only this classification, this study could not evaluate surgical rehabilitation based on other classifications, although they were more suitable. It is possible that the classification of CL/P at hospitalization was inaccurately recorded, especially considering that CL and CP surgeries might have been performed on people with CLP, but at different surgical times.

Regarding data collection in the SUS hospital information system, the free software Tab for Windows (TabWin®) version 4.15 was used. The surgical procedures collected in the study, due to varying types and names, needed to be grouped using the anatomical region mentioned in their nomenclature as a reference. Thus, the groups of procedures organized in the final analyses were lip surgery, palate surgery, nose surgery, dental alveolus surgery, ear or ear canal surgery, multiple surgery, and other procedures. The multiple surgery group represented the SUS technical record at the authorization for hospitalization when more than one surgical procedure was performed on a patient in the same anesthetic act. The public financial values were converted from the Brazilian currency into dollars using the annual quotation averages for each year of analysis.

The presence and type of CL/P were identified by the ICD-10 codes, which are Q35 (CP), Q36 (CL), and Q37 (cleft palate with cleft lip).^24^ Statistical analyzes were performed using the statistical program SPSS 16, version 24.0, assuming a significance level of 95% (*P* < .05). The bivariate analysis was performed using Pearson's correlation and the multivariate analysis using the Wilks' Lambda test. The financial value was calculated from the sum of the total values of the hospitalizations, adjusted by the dollar rate at the end of each year evaluated.

## Results

**Figure 1:**
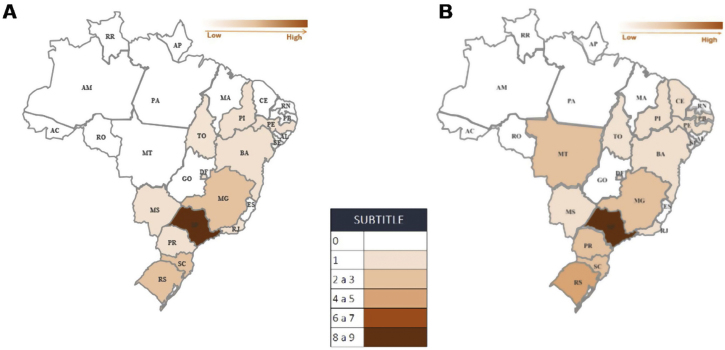
Geographic distribution of the enabled Lip and Palate Malformation Treatment Centers, by federative unit, with 22 in 2008 (A) and with 30 in 2020 (B).

In 2008, 22 treatment centers were authorized by the SUS, in 100% of the geographic regions and 44.4% of the Brazilian federative units (n = 12). In 2020, there was an increase of 36.4% (n = 8), with a total of 30 treatment centers authorized in 55.5% of the federative units (n = 15) (Fig. 1).

From 2008 to 2020, 68,716 surgical procedures for the treatment of CL/P were performed at the treatment centers, with an average of 5286 procedures per year. There was no significant variation in the number of surgical procedures performed between 2008 and 2019; however, in 2020, the country presented a reduction of 50.7% in the performance of CL/P surgeries compared to the average number from previous years, being more pronounced in the North (85% reduction) and South (61.5% reduction) (Table 1). In four treatment centers, no records of hospital surgical procedures for CL/P were identified.

**Table 1:** Procedures performed for surgical rehabilitation of cleft lip and/or palate by the Brazilian public health system in Treatment Centers for Malformation of the Lip and Palate, by year and Regions geographics.

Year of hospitalization	Brazil	Midwest	North	Northeast	Soulth	Soultheast
Average						
2008‒2019	5500	78.5	26.5	933.2	1160.3	3301.1
2020	2721	56	4	492	447	1722
Difference	2779	−28.6%	−84.9%	−47.3%	−61.5%	−52.2%

There was a significant difference in the surgery by geographic region, federative unit, and treatment centers. Surgical rehabilitation of CL/P occurred predominantly in the Southeast, South, and Northeast of Brazil, with emphasis on the Southeast region. The federative unit with the largest number of surgical procedures for CL/P was São Paulo and treatment centers, Craniofacial Anomaly Rehabilitation Hospital of the University of São Paulo.

**Figure 2:**
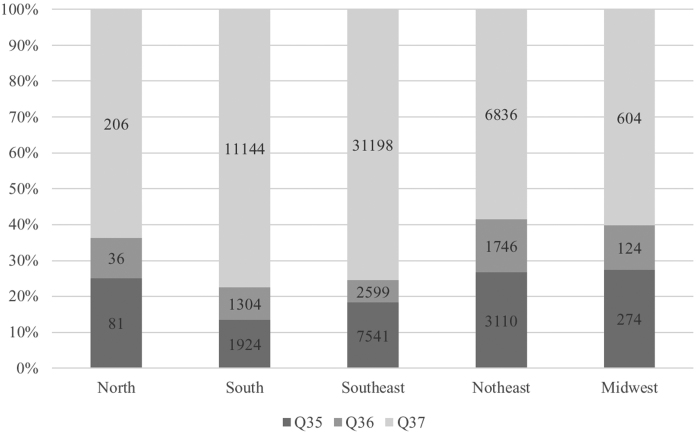
Percentage of hospitalizations by ICD-10^a^ code of cleft lip and/or palate by Brazilian public health system in Lip and Palate Malformation Treatment Centers, by geographic region, from 2008 to 2020 (*P* < .001). ^a^ International Statistical Classification of Diseases and Related Health Problems, 10th review.

**Figure 3:**
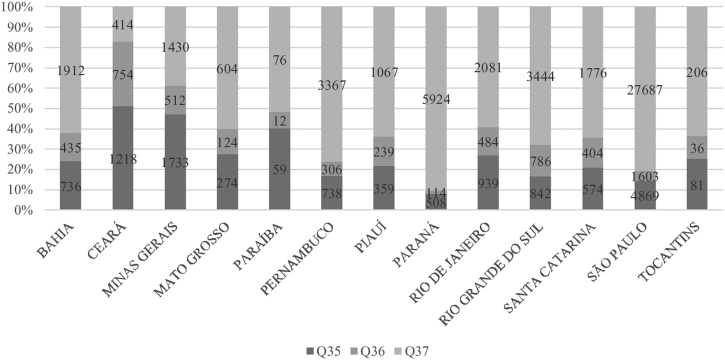
Number of hospitalizations by ICD-10^a^ code of cleft lip and/or palate by Brazilian public health system in Lip and Palate Malformation Treatment Centers, by federative unit^b^, from 2008 to 2020 (*P* < .001). ^a^ International Statistical Classification of Diseases and Related Health Problems,10th review. ^b^ The federative units that have LPMTC enabled by SUS are: Bahia (BA), Ceará (CE), Federal District (DF), Minas Gerais (MG), Mato Grosso do Sul (MS), Mato Grosso (MT), Pernambuco (PE), Piauí (PI), Paraná (PR), Rio de Janeiro (RJ), Rio Grande do Sul (RS), Santa Catarina (SC), São Paulo (SP) and Tocantins (TO).

Considering a variety of 85 surgical procedures performed, the most frequent were multiple surgeries. There was a significant difference between the types of surgeries by geographic region, federative unit, and treatment centers (*P* < .001). The most frequent ICD-10 codes in the hospitalizations evaluated were CLP. The predominance of CLP occurred in all geographic regions (*P* < .001) (Fig. 2) and in most federative units (Fig. 3).

**Figure 4:**
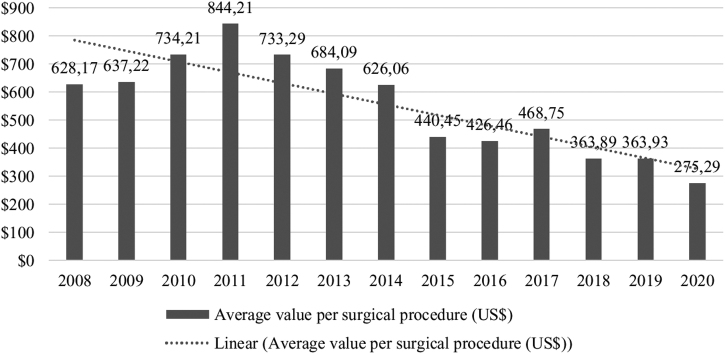
Average value per surgical procedure (in dollars) for surgical rehabilitation of cleft lip and/or palate by the Brazilian public health system in Lip and Palate Malformation Treatment Centers enabled, by year (*P* < .001).

Public financial resources invested in the surgical rehabilitation of CL/P in treatment centers were US$ 39,693 million, with an average of US$ 3053 million per year. Pearson's correlation revealed a significant difference between the years evaluated (*P* < .001). The mean value per surgical procedure for CL/P in Brazil was US$ 577.64, with the highest annual value of public investments in 2011 and an average value per surgical procedure of US$ 844,21 (Fig. 4).

## Discussion

Brazilian legislation defines treatment centers as health facilities with the physical structure, equipment, and specialized human resources to provide optimal clinical and surgical care to patients with CL/P in Brazil.^25^, ^26^ Although they are in all geographic regions of the country since 2020, 12 Brazilian federative units still do not have treatment centers authorized by the SUS. This situation may indicate that, in these regions, the population has to travel long distances to access qualified treatment, which may increase the delay to treatment initiation and public expenses, considering the large geographic expanse of Brazil.^27^, ^28^, ^29^

The predominance of surgical rehabilitation for CL/P in the Southeast, South, and Northeast of Brazil, especially in the Southeast, coincides with the results of other studies,^27^, ^30^ as well as the geographic distribution of treatment centers close to the country's higher education institutions.^27^ Monlleó and Gil-da-Silva-Lopes (2006) investigated the characteristics of 25 treatment centers up to 2003 and observed a predominance of services in the Southeast region, in universities, and with predominantly public funding.^27^ Sousa and Roncalli (2017) identified that the predominance of surgical rehabilitation for CL/P in the Southeast region indicates that the country needs better management and planning of hospital health services to meet the needs of individuals with CL/P by SUS.^23^

Although the surgical rehabilitation of CL/P in Brazil is not present throughout the national territory, Monlleó et al. (2009) observed that this service is also offered by a significant number of health units involved in the surgical rehabilitation of craniofacial anomalies but not authorized by the SUS.^30^ These units demonstrate overlapping with the services of treatment centers authorized by the SUS in many aspects, such as greater geographic distribution, presence of institutional affiliation, other funding sources, and more types of clefts treated, mainly represented by patients with CL/P. Thus, although not recognized by the SUS network, these alternative services may be offered treatment with greater ease of access, especially in regions where the presence of qualified centers is scarce.^30^

Concerning federative units, the expressive procedures in São Paulo, especially at the Hospital for Rehabilitation of Craniofacial Anomalies of the University of São Paulo, might have occurred because this treatment center was one of the first authorized by SUS and, for several years, it was a national and international reference center in the treatment of CL/P.^15^

The significant reduction in the number of surgical procedures performed in 2020 was influenced by the restriction actions caused by the Coronavirus Disease-2019 (COVID-19) pandemic, as the World Health Organization recommended that elective procedures be postponed in all countries with COVID-19 until the end of the table acute phase of the pandemic.^24^ Thus, access reduction measures were implemented to reduce elective access, maintaining only emergencies, to contain the spread of the virus, and it is possible that this reduction impacted the rehabilitation and monitoring of individuals with CL/P in the country and worldwide.^31^

Multiple surgeries as the most frequent procedure may demonstrate that individuals with CL/P arrive at treatment centers, requiring more interventions, involving several anatomical structures in the same anesthetic act, indicating greater complexity of the CL/P. This finding is in accordance with the prevalence studies in Brazil of complete clefts involving the lip and palate,^13^, ^23^ as well as with the results of the study by Paranaíba et al. (2009), who demonstrated a higher frequency of combined surgical techniques when compared to isolated procedures.^32^ The results may also indicate that individuals accessed the treatment centers late, presenting a greater need for surgical interventions since the delay at the beginning of the surgical treatment of CL/P in Brazil is a reality identified by other researchers.^23^, ^33^

Surgical techniques for the correction of CLP can be one-stage, with the closure of the lip and palate together in the same anesthetic act or two stages, with the initial closure of the lip and after the palate. Although other studies that analyzed the two techniques did not observe a significant difference between them with regard to the patient's bone impact,^34^, ^35^ these two types of surgical techniques may have influenced the results of this study, either by the increase in the number of multiple surgeries when the single-stage technique was used or by the increase in palate surgeries in relation to labial surgeries, if the two-stage technique was used. In the present study, it is not possible to state which surgical techniques were used.

In contrast to the current prevalence of CL/P in Brazil, which shows a higher frequency of CL compared to CP,^13^, ^23^ the higher number of isolated palate surgeries in relation to isolated lip may be related to the fact that isolated lip surgeries are not being referred for surgeries in qualified centers due to their lesser surgical complexity^36^; thus, patients with CL only may prefer outpatient clinics or local hospitals instead of the center qualified for treatment.^30^

Regarding the public financial resources invested in the surgical treatment of CL/P in the treatment centers, it was observed that the devaluation of the Brazilian currency against the dollar over the years evaluated generated a gradual reduction in the data on annual amounts paid. The total value of US$ 37,903 million represents less than 1% of Brazil's Gross Domestic Product (GDP), which was US$ 19,029 trillion in the same period.^37^ Saldiva and Veras (2018) observed that general public health financing in Brazil has fluctuated by approximately 8% of the GDP in recent years and that other countries that offer universal access to health spend slightly more resources than Brazil, such as Canada (10.4% of the GDP) and the United Kingdom (9.9% of the GDP),^38^ indicating that in Brazil expenditure may be inefficient and perhaps there is no real underfunding of the health system.^39^, ^40^

The analysis of the cost of surgical treatment for CL/P is still a divergent point due to the methods used and the items that are either included in the sum or not, such as hospital costs, payment of professionals, drugs, tests, among others.^41^ In Brazil, each surgical procedure has a value set by SUS that includes hospital and professional services.^42^ The average cost of surgical treatment for CL/P by SUS in Brazil identified in the present study (US$ 577.64) was lower than that estimated in developing countries, which presented values close to US$ 800 for CL/P surgery performed by non-governmental organizations.^28^, ^41^ In the United States of America, which has private health financing, a study estimated the cost of a day of hospitalization for isolated CL surgery at US$ 2,390, and in Canada, where there is a public health system, at US$ 1695.^43^ The value identified in this study may have been influenced when the Brazilian currency was converted to the value of each year's dollar.

Little is known regarding the measurement of the costs of surgical rehabilitation of CL/P in Brazil. Considering congenital malformations as a whole, Horovitz et al. (2005) identified that the average cost of hospitalization for congenital malformations in Brazil was US$ 400.09 in 2002, showing a significant difference from the average value of US$ 125.18 for general hospitalization.^44^

Regarding the geographic distribution of public financial resources allocated to the surgical treatment of CL/P, Brazil exhibited a polarization in the Southeast region, where the centers that have been qualified for the longest time in the country are located. Brazil, as a large, populous, and geographically diverse country, may have locoregional differences and thus face several influences in the expansion of the SUS network and the access of individuals with CL/P to surgical rehabilitation in the treatment centers authorized by the SUS.^23^, ^30^

The limitations of this study regarding the data collected are related to the possibility that they do not represent all surgeries performed for CL/P by SUS, since other non-qualified public and private services can also perform the surgeries. There is a possibility of underreporting of LF/P cases in Brazil. The limitations of this study regarding the financial analysis are related to the fact that the data collected may not have represented all the amounts transferred by the SUS for the treatment of CL/P, and there may be other sources of funding for the qualified centers. Furthermore, during the conversion of the Brazilian currency to the dollar, the analysis was influenced by the increase in the dollar in the evaluated period, causing the impression of a reduction in values over time, which may not have actually occurred.

## Conclusion

In Brazil, the SUS carried out and financed over the years 2008 and 2020, an important number of surgical procedures for CL/P, which presented a polarization of care in the Southeast region. The most common surgical procedures were multiple surgeries, mainly in individuals with complete CLP. The results of this study cannot state that the heterogeneity in the geographic distribution of these services in Brazil may influence the type of procedures performed and the cracks attended to, requiring further studies for this statement. Considering that the surgical rehabilitation of CL/P is an initial and fundamental step for adequate assistance to the individual, impacting on their morbidity, mortality, and quality of life, it is understood that it should, therefore, be the object of special attention in the planning, implementation, and evaluation of the policy of attention to CL/P in Brazil, especially in the context of the current pandemic.

## Conflicts of interest

The authors declare no conflicts of interest.

## References

[1] Gil-da-Silva-Lopes V.L., Monlleó I.L. (2014). Risk factors and the prevention oral clefts. Braz Oral Res..

[2] Abu-Hussein M., Watted N., Hegedűs V., Borbély P., Azzaldeen A. (2015). Human genetic factors in non-syndromic cleft lip and palate: an update. I J Maxilofac Res..

[3] Lidral A.C., Moreno L.M., Bullard S.A. (2008). Genetic factors and orofacial clefting. Semin Orthod..

[4] Voigt A., Radlanski R.J., Sarioglu N., Schmidt G. (2017). Cleft lip and palate. Pathologe..

[5] Machado R.A., Freitas E.M., Aquino S.N., Martelli D.R.B., Swerts M.S.O., Reis S.R. (2017). Clinical relevance of breast and gastric cancer-associated polymorphisms as potential susceptibility markers for oral clefts in the Brazilian population. BMC Med Genet..

[6] Watkins S.E., Meyer R.E., Strauss R.P., Aylsworth A.S. (2014). Classification, epidemiology, and genetics of orofacial clefts. Clin Plast Surg..

[7] Shrestha A., Takahashi M., Yamaguchi T., Adel M., Furuhata M., Hikita Y. (2020). Three-dimensional evaluation of mandibular volume in patients with cleft lip and palate during the deciduous dentition period. Angle Orthod..

[8] Dixon M.J., Marazita M.L., Beatyt H., Murray J.C. (2011). Cleft lip and palate: synthesizing genetic and environmental influences. Nat Rev Genet..

[9] Silva H.P.V., Arruda T.T.S., Souza K.S.C., Bezerra J.F., Leite G.C.P., Brito M.E.F. (2018). Risk factors and comorbidities in Brazilian patients with orofacial clefts. Braz Oral Res..

[10] Machado R.A., Silva C.O., Persuhn D.C., Dantas V.M.C., Reis S.R.A., Wu T. (2019). Interactions between superoxide dismutase and paraoxonase polymorphic variants in nonsyndromic cleft lip with or without cleft palate in the brazilian population. Environ Mol Mutagen..

[11] Cobourne M. (2004). The complex genetics of cleft lip and palate. Eur J Orthod..

[12] Vieira A.R. (2008). Unraveling human cleft lip and palate research. J Dent Res..

[13] Martelli Junior H., Porto L.V., Martelli D.R.B., Bonan P.R.F., Freitas A.B., Coletta R.D. (2007). Prevalence of non-syndromic oral clefts in a reference hospital in the state of Minas Gerais, Brazil, between 2000-2005. Braz Oral Res..

[14] Rodrigues K., Sena M.F., Roncalli A.G., Ferreira M.A.F. (2009). Prevalence of orofacial clefts and social factors in Brazil. Braz Oral Res..

[15] Freitas J.A.S., Neves L.T., Almeida A.L., Garib D.G., Trindade-Suedam I.K., Yaedú R.Y. (2012). Rehabilitative treatment of cleft lip and palate: experience of the Hospital for Rehabilitation of Craniofacial Anomalies/USP (HRAC/USP) – Part 1: overall aspects. J Appl Oral Sci..

[16] American Cleft Palate-Craniofacial Association (ACPA) (2018). Parameters for evaluation and treatment of patients with cleft lip/palate or other craniofacial differences. Cleft Palate Craniofac J..

[17] Brazil. Ministry of Health. Ordinance SAS/MS nº 126, of September 17, 1993. Creates groups and procedures for the treatment of cleft lip and palate in the SIH/SUS table, and other measures. Officer of the Federative Republic of Brazil, Brasília/DF, 1993. Available at: http://sna.saude.gov.br/legisla/legisla/alta_lab_p/. Accessed November 30, 2021.

[18] Brazil. National Council of Health Secretaries. SUS: advances and challenges. Brasília/DF, 2006. Available at: https://www.conass.org.br/bibliotecav3/pdfs/Livro_Sus.pdf. Accessed November 30, 2021.

[19] Rando G.M., Jorge P.K., Vitor L.L.R., Carrara C.F.C., Soares S., Silva T.C. (2018). Oral health-related quality of life of children with oral clefts and their families. J Appl Oral Sci..

[20] Silva M.A.R., Balderrama I.F., Wobeto A.P., Werneck R.I., Azevedo-Alanis L.R. (2018). The impact of nonsyndromic cleft lip with or without cleft palate on oral health-related quality of life. J Appl Oral Sci..

[21] Poenaru D., Lin D., Corlew S. (2016). Economic valuation of the global burden of cleft disease averted by a large cleft charity. World J Surg..

[22] Saxton A.T., Poenaru D., Ozgediz D., Ameh E.A., Farmer D., Smith E.R. (2016). Economic analysis of children’s surgical care in low- and middle-income countries: a systematic review and analysis. PLoS One..

[23] Sousa G.F.T., Roncalli A.G. (2017). Orofacial clefts in Brazil and surgical rehabilitation under the Brazilian National Health System. Braz Oral Res..

[24] World Health Organization (WHO). International Statistical Classification of Diseases and Related Health Problems, 10th revision, 2008. Available at: http://www.datasus.gov.br/cid10/V2008/cid10.htm. Accessed November 30, 2021.

[25] Brazil. Ministry of Health. Ordinance SAS/MS nº. 62, April 19, 1994. Establishes the rules for the registration of hospitals that perform integrated procedures for aesthetic-functional rehabilitation of patients with lip and palate cleft malformation for the Unified Health System. Officer of the Federative Republic of Brazil, Brasília/DF, 1994. Available at: https://bvsms.saude.gov.br/bvs/saudelegis/sas/1994/prt0062_19_04_1994.html. Accessed November 30, 2021.

[26] Brazil. Ministry of Health. Secretariat of Health Care. Department of Regulation, Evaluation and Control. Manual of the National Register of Health Establishments/CNES. 2th version. Brasília/DF, 2006. Available at: https://jundiai.sp.gov.br/saude/wp-content/uploads/sites/17/2014/09/Manual-de-Preenchimento-SCNES-Fichas-completas.pdf. Accessed November 30, 2021.

[27] Monlleó I.L., Gil-Da-Silva-Lopes V.L. (2006). Craniofacial anomalies: description and evaluation of treatment under the Brazilian Unified Health System. Cad Saude Publica..

[28] Magee W.P., Burg R.V., Hatcherk W. (2010). Cleft lip and palate as a cost-effective health care treatment in the developing world. World J Surg..

[29] Mossey P.A., Shaw W.C., Munger R.G., Murray J.C., Murthy J., Little J. (2011). Global oral health inequalities: challenges in the prevention and management of orofacial clefts and potential solutions. Adv Dent Res..

[30] Monlleó I.L., Mossey P.A., Gil-Da-Silva-Lopes V.L. (2009). Evaluation of craniofacial care outside the brazilian reference network for craniofacial treatment. Cleft Palate Craniofac J..

[31] Alexandre L.P., Cançado L.N.L., Pretti H., Lages E.M.B., Lima Y.C.F., Pascoaloti M.I.M. (2022). Importance of the treatment of patients with lip and palate cleft, especially during the COVID-19 pandemic. Oral Surg..

[32] Paranaíba L.M.R., Almeida H., Barros L.M., Martelli D.R.B., Orsi-Júnior J.D., Martelli-Júnior H. (2009). Current surgical techniques for cleft lip-palate in Minas Gerais, Brazil. Braz J Otorhinolaryngol..

[33] Ise A., Menezes C., Batista-Neto J., Saluja S., Amundson J.R., Jenny H. (2019). Patient-Perceived Barriers to Accessing Cleft Care at a Tertiary Referral Center in São Paulo, Brazil. Cleft Palate Craniofac J..

[34] Stein S., Dunsche A., Gellrich N.C., Harle F., Jonas I. (2007). One- or two-stage palate closure in patients with unilateral cleft lip and palate: comparing cephalometric and occlusal outcomes. Cleft Palate Craniofac J..

[35] Tome W., Yashiro K., Otsuki K., Kogo M., Yamashiro T. (2016). Influence of different palatoplasties on the facial morphology of early mixed dentition stage children with unilateral cleft lip and palate. Cleft Palate Craniofac J..

[36] Figueiredo I.M.B., Bezerra A.L., Marques A.C.L., Rocha I.M., Monteiro N.R. (2004). Surgical treatment of complete cleft palate. Rev Bras Promoção Saúde..

[37] World Bank Group. World Development Indicators. Available at: https://databank.worldbank.org/source/world-development-indicators. Accessed November 30, 2021.

[38] Saldiva P.H.N., Veras M. (2018). Public spending on health: brief history, current situation and future perspectives. Estud av..

[39] Piola S.F., Paiva A.B., Sá E.B., Servo L.M.S. (2013). http://hdl.handle.net/10419/91380.

[40] Giovanella L., Stegmuller K. (2014). The financial crisis and health care systems in Europe: universal care under threat? Trends in health sector reforms in Germany, the United Kingdom, and Spain. Cad Saude Publica..

[41] Sheriff S., Zawahrah H.J., Chang L.V., Beyatli S., Babiker H.M.E., Roach A.L. (2018). What is the cost of free cleft surgery in the middle east?. World J Surg..

[42] Brazil. Ministry of Health. Management System of Table of Procedures, Drugs, Orthoses, Prostheses and Materials of the SUS. Brazil: 2021. Available at: http://sigtap.datasus.gov.br/tabela-unificada/app/sec/inicio.jsp. Accessed November 30, 2021.

[43] Arneja J.S., Mitton C. (2013). Ambulatory cleft lip surgery: a value analysis. Can J Plast Surg..

[44] Horovitz D.D.G., Llerena J.C., Mattos R.A. (2005). Birth defects and health strategies in Brazil: an overview. Cad Saude Publica..

